# Liquid biopsy detects genomic drivers in NSCLC without *EGFR* mutations by single‐plex testing: WJOG13620L

**DOI:** 10.1002/cam4.6668

**Published:** 2023-11-10

**Authors:** Takehiro Uemura, Hirotsugu Kenmotsu, Daisuke Hazama, Shunsuke Teraoka, Hiroshi Kobe, Koichi Azuma, Teppei Yamaguchi, Takeshi Masuda, Toshihide Yokoyama, Kohei Otsubo, Koji Haratani, Daisuke Hayakawa, Masahide Oki, Shinnosuke Takemoto, Tomohiro Ozaki, Yusaku Akashi, Akito Hata, Hiroya Hashimoto, Nobuyuki Yamamoto, Kazuhiko Nakagawa

**Affiliations:** ^1^ Department of Respiratory Medicine, Allergy and Clinical Immunology Nagoya City University Graduate School of Medical Sciences Nagoya Aichi Japan; ^2^ Division of Thoracic Oncology Shizuoka Cancer Center Shizuoka Japan; ^3^ Division of Respiratory Medicine Department of Internal Medicine Kobe University Graduate School of Medicine Kobe Hyogo Japan; ^4^ Internal Medicine III Wakayama Medical University Wakayama Wakayama Japan; ^5^ Department of Respiratory Medicine Kobe City Medical Center General Hospital Kobe Hyogo Japan; ^6^ Division of Respirology, Neurology, and Rheumatology, Department of Internal Medicine Kurume University School of Medicine Fukuoka Japan; ^7^ Department of Thoracic Oncology Aichi Cancer Center Nagoya Aichi Japan; ^8^ Department of Respiratory Medicine Hiroshima University Hospital Hiroshima Japan; ^9^ Department of Respiratory Medicine Kurashiki Central Hospital Okayama Japan; ^10^ Department of Respiratory Medicine Kitakyushu Municipal Medical Center Fukuoka Japan; ^11^ Department of Medical Oncology Kindai University Faculty of Medicine Osaka Japan; ^12^ Department of Respiratory Medicine Juntendo University, Graduate School of Medicine Tokyo Japan; ^13^ Department of Respiratory Medicine National Hospital Organization Nagoya Medical Center Nagoya Aichi Japan; ^14^ Department of Respiratory Medicine Nagasaki University Graduate School of Biomedical Sciences Nagasaki Japan; ^15^ Department of Medical Oncology Kishiwada City Hospital Osaka Japan; ^16^ Department of Medical Oncology Kindai University Nara Hospital Nara Japan; ^17^ Department of Thoracic Oncology Kobe Minimally Invasive Cancer Center Kobe Hyogo Japan; ^18^ Clinical Research Management Center Nagoya City University Hospital Nagoya Aichi Japan

**Keywords:** circulating tumor DNA, liquid biopsy, lung cancer, molecularly targeted therapy, multiplex gene analysis

## Abstract

**Background:**

Actionable tumor genomic alterations, primarily *EGFR* mutations, occur in nearly 70% of Japanese advanced nonsquamous non‐small cell lung cancer (NSCLC) patients. Standard assessment of tumor tissue includes rapid testing for *EGFR* mutations, *ALK* fusions and *ROS1* fusions. We conducted a prospective observational study (WJOG13620L) of follow‐on next‐generation sequencing of circulating tumor DNA (ctDNA) in patients without driver alterations after *EGFR* testing.

**Methods:**

Patients with untreated advanced (Stage IIIB–IV or relapsed) nonsquamous NSCLC without *EGFR* mutations according to single‐plex testing of tumor tissue, were enrolled into this study. Patients with other known driver mutations or who underwent comprehensive genomic profiling were excluded. Plasma was analyzed by Guardant360, and the primary endpoint was the proportion of patients with pathogenic gene alterations in at least one of nine genes.

**Results:**

Among the 72 patients enrolled, *ALK* and *ROS1* fusions were tested in 86.1% and 65.2%, respectively. Alterations in pre‐defined genes were detected in 21 patients (29.2%; 95% confidence interval: 19.0–41.1, *p* < 0.001 [one‐sided null hypothesis proportion of 10%]), including *RET* fusion (*n* = 1) and mutations in *KRAS* (*n* = 11), *EGFR* (*n* = 5), *ERBB2* (*n* = 3), and *BRAF* (*n* = 1). Median time from sample submission to results was 8 days (range, 5–17 days).

**Conclusion:**

Rapid follow‐on comprehensive testing of ctDNA should be considered prior to first‐line treatment for patients with advanced nonsquamous NSCLC when no alterations are detected after single‐plex tissue testing.

## Graphical abstract

Alterations in pre‐defined nine genes were detected in 21 NSCLC patients by multiplex gene analysis.

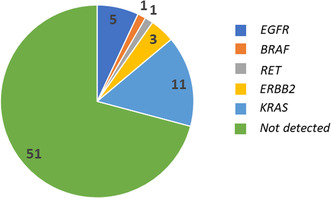

## 1 INTRODUCTION

Current National Comprehensive Cancer Network guidelines recommend that all patients with newly diagnosed advanced lung adenocarcinoma should be tested for driver alterations, as tumors harboring these driver oncogene alterations can be treated with molecularly targeted therapies.^1^ Targeted drugs for *EGFR*, *ALK*, *ROS1*, *BRAF*, *RET*, *MET*, *KRAS* and *NTRK* alterations have already been approved in Japan and corresponding molecular tests have been introduced to clinical practice simultaneously with approval of the respective molecularly targeted drugs. Each test requires a sufficient tissue sample, whereas specimens collected from advanced non‐small cell lung cancer (NSCLC) patients are often either small or cytology specimens. Conventional single‐plex gene tests have been performed to identify patients who may be responsive to molecularly targeted drugs. However, as the number of identified driver oncogene alterations increases, more genetic tests are required to determine treatments for advanced NSCLC.^2^, ^3^, ^4^, ^5^, ^6^, ^7^, ^8^, ^9^ Given this background, multiplex genetic analysis is highly advantageous in clinical practice, enabling the analysis of limited amounts of biopsied tissue samples.

In Japan, participation in the national health insurance system is mandatory and covers most medical expenses. Under this system, medical doctors request the use of medical procedures, equipment and drugs that have been approved by the Japanese Ministry of Health, Labour and Welfare.^10^, ^11^ Support for treatment with the approved precision medicine requires the use of an approved assay for the detection of the relevant biomarker. Although multi‐gene assays are approved for assessing most actionable biomarkers prior to first‐line treatment, single‐plex gene analysis is still used in patients with limited tissue samples. In the absence of accurate identification, opportunities for implementing appropriate molecularly targeted therapies against advanced NSCLC may be missed. On the other hand, repeated biopsies may not be feasible for many patients because of anatomical difficulties, existing comorbidities, and/or clinical deterioration forcing rapid initiation of medical treatment.

In daily practice, cases are sometimes encountered in which only some driver genes can be tested because of insufficient or unavailable tissue samples. In this case, multiplex gene analysis of circulating tumor DNA (ctDNA) may be useful. To verify the utility of ctDNA next‐generation sequencing (NGS) in such situations, we planned this prospective study to assess the clinical performance of ctDNA NGS in a multi‐institutional prospective cohort of patients diagnosed with advanced‐stage nonsquamous NSCLC who had been tested for at least *EGFR* mutations by single‐plex gene analysis and were negative for any driver alterations.

## 2 MATERIALS AND METHODS

### 2.1 Study design and patients

This prospective observational study (WJOG13620L/STARLIGHT) evaluated the detection rate of actionable gene alterations by ctDNA NGS in patients with nonsquamous NSCLC for whom gene alterations were not detected in tissue‐based single‐plex assays. This multicenter, prospective study recruited patients at 24 institutions in Japan. Patients were enrolled if they met the following eligibility criteria: (1) histologically confirmed nonsquamous NSCLC; (2) Stage IIIB–IV or recurrent disease after surgery or chemoradiotherapy; (3) age ≥ 20 years; (4) *EGFR* gene confirmed as wild type by single‐plex assay and “unknown” or “negative” results for the other eight genes (*ALK*, *ROS1*, *BRAF*, *KRAS*, *MET*, *RET*, *ERBB2*, and *NTRK*) tested at the time of enrollment; (5) comprehensive genomic profiling had not been performed; (6) no prior treatment with systemic anti‐cancer treatment (e.g., cytotoxic agent, molecularly targeted therapy, or immune checkpoint inhibitor) for advanced‐stage NSCLC (excluding postoperative adjuvant chemotherapy or chemoradiotherapy); (7) disease considered treatable with anti‐cancer drugs; (8) life expectancy ≥3 months; and (9) written informed consent from the patient after sufficient explanation of the study. The key exclusion criteria were as follows: (1) presence of other malignancies with metastases; (2) serious mental disease; and (3) not fit for this trial as determined by the treating physician.

This study was conducted in accordance with the Declaration of Helsinki and the Japanese ethical Guidelines for Medical and Health Research Involving Human Subjects. The study protocol was approved by the institutional review board of each participating institution and registered at the University Hospital Medical Information Network Clinical Trial Registry (protocol no. UMIN000041583).

### 2.2 Blood samples, ctDNA isolation, and ctDNA sequencing

The NGS analysis of ctDNA was conducted using Guardant360 (Guardant Health, Redwood City, CA) at Guardant Health, a Clinical Laboratory Improvement Amendments‐certified, College of American Pathologist‐accredited, New York State Department of Health‐approved assay, as previously described.^12^ This assay interrogates 74 genes and detects single‐nucleotide variants (SNVs), insertions and deletions, fusions, and copy number alterations. Whole blood (20 mL) was collected in Streck tubes (Streck, La Vista, Nebraska) during routine phlebotomy before starting first‐line treatment, and samples were shipped at ambient temperature overnight to California from Japan. Analyzed results were promptly reported to physicians via an electronic portal site.

### 2.3 Data analysis

Patient backgrounds were evaluated for all enrolled patients. The primary endpoint of this study was the proportion of patients with an alteration found by ctDNA NGS in any one of nine clinically relevant genes: *EGFR*; *ALK*; *ROS1*; *BRAF*; *KRAS*; *MET*; *RET*; *ERBB2*; and *NTRK*. We set the clinically useful discovery rate (H_1_) as 25%, roughly half the value reported in a prior study using a tissue‐based follow‐on multiplex assay.^13^ We set the threshold rate (H_0_) as 10% because the test seems to be clinically useful if the multiplex test finds about 10% of driver gene alterations in patients for whom no driver gene alterations were found by the single‐plex test. The required sample size was calculated as 65 patients using a binomial test (one‐sided α = 0.025, β = 0.10). The planned total sample size was set at 72 considering the potential ineligibility of patients or inadequate quality of samples. The two‐tailed 95% confidence interval (CI) was used to evaluate statistical significance. Secondary endpoints were the percentages of patients with each of the nine major alterations detected, turnaround time (TAT), rate of patients showing gene alteration detected using companion diagnostic (CDx) methods to confirm the gene alteration detected in ctDNA, and the rate of patients treated with molecularly targeted therapies based on the ctDNA result, up to 6 months after study enrollment. We defined TAT as the period from the day blood was collected to the day that the ctDNA result was obtained from the website. All statistical analyses were performed using SAS version 9.4 (SAS Institute, Cary, NC, USA).

## 3 RESULTS

### 3.1 Patient characteristics

In total, 72 patients were enrolled between October 2020 and May 2021. The baseline characteristics of patients are summarized in Table 1. Median age was 72.5 years (range, 47–86 years) and 50 patients (69.4%) were male. Sixteen patients (22.2%) were never‐smokers, whereas 56 (76.4%) patients had a history of smoking. The performance status of these patients was generally 0 or 1 (91.6%). Percentages of patients with adenocarcinoma and with Stage IV disease were 91.7% and 73.6%, respectively.

**TABLE 1:** Characteristics of the 72 patients in the present study.

Age (years)		
Median	72.5	%
Range	47–86	
Sex		
Male	50	69.4
Female	22	30.6
Smoking history		
Never	16	22.2
Past	38	52.8
Current	17	23.6
Unknown	1	1.4
Performance status		
0	23	31.9
1	43	59.7
2	6	8.3
Pathology		
Adenocarcinoma	66	91.7
Large cell carcinoma	2	2.8
Sarcomatoid carcinoma	2	2.8
Other	2	2.8
Stage		
IIIB	3	4.2
IIIC	3	4.2
IVA	16	22.2
IVB	37	51.4
Postoperative recurrence	8	11.1
Post‐chemoradiotherapy recurrence	5	6.9
T stage		
x	2	2.8
0	2	2.8
1	18	25
2	14	19.4
3	19	26.4
4	17	23.6
N stage		
x	1	1.4
0	11	15.3
1	8	11.1
2	21	29.2
3	31	43
M stage		
0	15	20.8
1a	13	18.1
1b	10	13.9
1c	34	47.2

### 3.2 Results of single‐plex gene analysis before plasma‐based NGS assay

**FIGURE 1:**
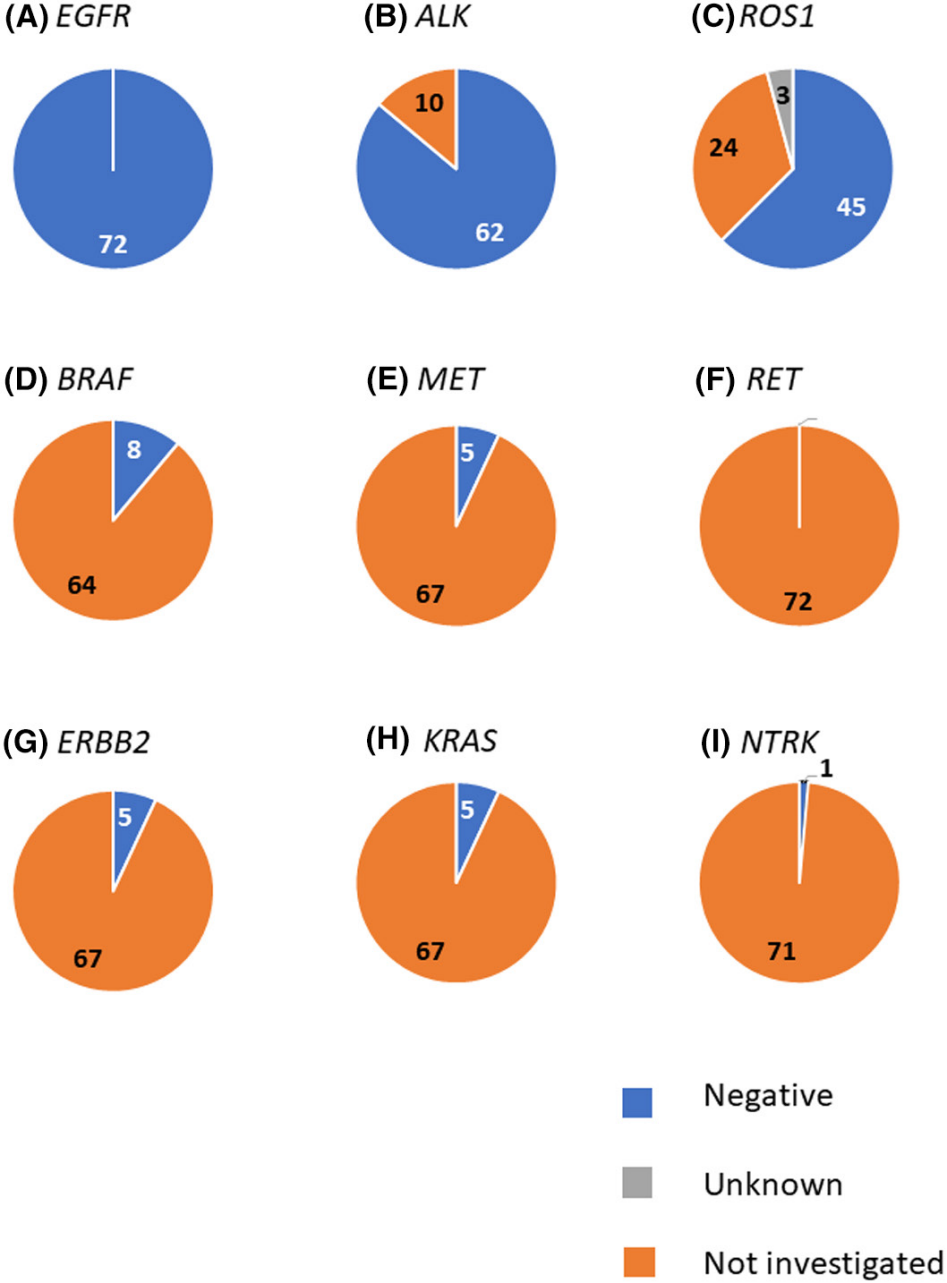
Results of tissue‐based single‐plex analysis before registration. In this study, the key inclusion criterion was that the *EGFR* gene was confirmed as the wild type by single‐plex assay. Circles indicate: (1) *EGFR*; (2) *ALK*; (3) *ROS1*; (4) *BRAF*; (5) *MET*; (6) *RET*; (7) *ERBB2*; (8) *KRAS*; and (9) *NTRK*. Blue, negative; gray, unknown; orange, not investigated.

All 72 patients enrolled had been tested for at least *EGFR* mutations by single‐plex gene analysis and showed negative results for any driver alteration (Figure 1). *ALK* fusion and *ROS1* fusion were tested in 62 (86.1%) and 42 (65.2%) of patients, respectively, whereas *BRAF* mutation, *MET* exon 14‐skipping mutation, *ERBB2* mutation, *KRAS* mutations, *NTRK* fusion, and *RET* fusion were only tested in 8 (11.1%), 5 (6.9%), 5 (6.9%), 5 (6.9%), 1 (1.4%), and 0 patients, respectively. A summary of complete ctDNA results for all 72 patients is shown in Table 2.

**TABLE 2:** Gene alterations detected in ctDNA. Alterations were found in 65 of 72 patients tested in 45 different genes.

Single nucleotide variant															
Gene name	*TP53*			*KRAS*	*EGFR*	*ARID1A*	*ATM*	*BRCA1*	*BRAF*	*AR*	*MET*	*PDGFRA*	*STK11*	*TERT*	
*n*	50			14	12	9	7	7	6	5	5	5	5	5	
	A138P	L111P	S127P	G12A	G322G	E1836*	I232F	I815I	G469A	G624V	D1380D	A292A	F157V	Promoter SNV	
	A138V	L194P	S183*	G12A	H773L	H210D	R1618Q	L1414L	K601E	L561Q	N382Y	L112L	Q214*	Promoter SNV	
	A159P	P278S	S241F	G12C	H1129Y	L1688*	R2832H	Q1721Q	K601E	P672P	P351L	L825L	W239*	Promoter SNV	
	A159V	R158L	S94*	G12C	L448L	M432I	R3008C	T1548M	K687Q	Q98K	R739C	R487L	Splice Site SNV	Promoter SNV	
	C135Y	R158L	V157F	G12D	L747P	Q268*	S1165T	T1691R	P192P	S29S	V264D	V272M	Splice Site SNV	Promoter SNV	
	C242F	R175H	V172D	G12D	L858R	Q479*	G3030V	V1341A	V600E						
	E180K	R175H	V173L	G12D	P564P	Q528*	Y583C	V162V							
	E204*	R248Q	V203L	G12F	R680R	S258C									
	E285K	R248Q	V216M	G12S	R973Q	S261W									
	E298*	R248Q	V272M	G12S	V774M										
	F134C	R248W	W53*	G12V	Y1016C										
	G154V	R249S	Y220C	G13D	Y813C										
	G199V	R249W	Y220N	V125V											
	H178D	R267W	Splice Site SNV	Y96C											
	I195T	R273H	Splice Site SNV												
	L111M	R273H	Splice Site SNV												
	L111P	R273L													
	*ALK*	*BRCA2*	*CDKN2A*	*APC*	*NOTCH1*	*PTEN*	*ROS1*	*SMAD4*	*CDK12*	*CTNNB1*	*ERBB2*	*IDH1*	*KIT*	*MAP2K1*	*MAPK3*
	4	4	4	3	3	3	3	3	2	2	2	2	2	2	2
	E1558Q	G25*	A60E	G2069S	D2091Y	Q171*	A2106T	R361C	M821T	S33F	R100W	R132C	N293S	D67Y	F348F
	F1480L	K1132R	A68P	T1459S	P2475P	R189K	D1966G	R445*	S283L	S37F	Y772Y	R132H	Splice Site SNV	R108Q	L129L
	M1290I	Q2157E	D108Y	T2820T	T2090T	Y188*	T1930I	Y95N							
	V979L	T2214A	R58*												
	*VHL*	*CCND1*	*CDK6*	*DDR2*	*ESR1*	*FGFR1*	*FGFR3*	*GATA3*	*GNAS*	*IDH2*	*MYC*	*NRAS*	*NTRK1*	*NTRK3*	*PIK3CA*
	2	1	1	1	1	1	1	1	1	1	1	1	1	1	1
	N67T	S225S	V205F	G809R	R300C	G260V	P402Q	L355L	R201H	M131I	C223S	R164C	R682C	R582W	I143T
	N150S														

Insertion and deletion.														
Gene name	*TP53*	*ERBB2*	*RB1*	*CDKN2A*	*EGFR*	*ARID1A*	*BRCA2*	*CDK12*	*CDK6*	*DDR2*	*MET*	*NOTCH1*	*PTEN*	*STK11*
*n*	10	4	3	2	2	1	1	1	1	1	1	1	1	1
	A86fs	A775_G776insYVMA	A525fs	V82fs	N771_H773dup	S263del	E2641fs	G141fs	F164fs	C651fs	V989fs	V2536fs	E235fs	R75fs
	N131del	A775_G776insYVMA	K722fs	c.137_150 + 14del	E709_T710delinsD									
	N210fs	A775_G776insYVMA	V87fs											
	P153fs	L823del												
	P177_C182del													
	S127del													
	T150fs													
	T155_R158del													
	V172_E180del													
	V97fs													

Copy number alteration							
Gene name	*CCNE1*	*BRAF*	*FGFR1*	*EGFR*	*CCND1*	*PDGFRA*	*PIK3CA*
*n*	6	2	2	2	1	1	1

Fusion		
Gene name	*FGFR3‐TACC3*	*KIF5B‐RET*
*n*	1	1

### 3.3 Detection rates of clinically relevant alterations by ctDNA NGS

**FIGURE 2:**
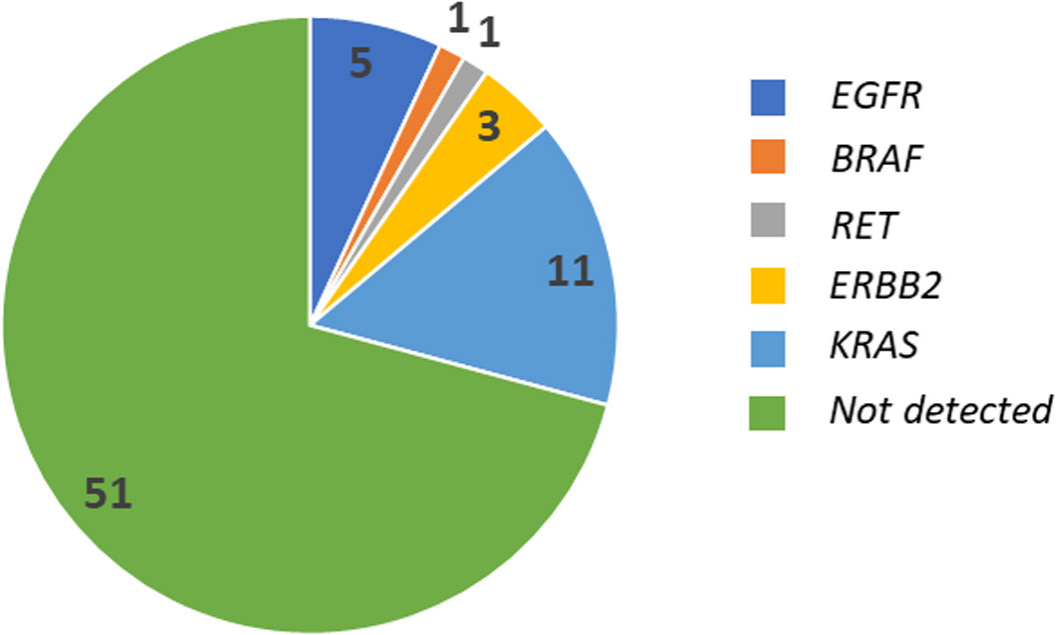
Detection rates for the nine driver‐gene alterations detected by multiplex gene analysis. Pie chart shows the number of cases. No driver alterations were detected in *NTRK*, *ROS1*, *ALK*, or *MET*.

The detection rate for alterations in at least one of the pre‐specified clinically relevant genes (*EGFR*, *ALK*, *ROS1*, *BRAF*, *MET*, *RET*, *KRAS*, *ERBB2*, and *NTRK*), as the primary endpoint, was 29.2% (*p* = 0.001; 95% CI, 19.0%–41.1%), which met the study hypothesis. Detection rates of alterations in each gene were as follows: 15.3% (*n* = 11; 95% CI, 7.9%–25.7%) for *KRA*S, 6.9% (*n* = 5; 95% CI, 2.3%–15.5%) for *EGFR*, 4.2% (*n* = 3; 95% CI, 0.9%–11.7%) for *ERBB2*, 1.4% (*n* = 1; 95% CI, 0%–7.5%) for *BRAF*, and 1.4% (*n* = 1; 95% CI, 0%–7.5%) for *RET* (Figure 2). Among the five patients with an *EGFR* mutation, the following mutations were found in one patient each: deletion in exon 18; L747P in exon 19; V774M/H773L in exon 20; insertion in exon 20; and L858R in exon 21. Among 11 patients with *KRAS* mutation, detection rates for the variants of *KRAS* mutation were as follows: 4.2% (*n* = 3) for G12D in exon 2; 2.8% (*n* = 2) for G12C in exon 2; 1.4% (*n* = 1) for G12S/G12A in exon 2; 1.4% (*n* = 1) for G12A in exon 2; 1.4% (*n* = 1) for G12F in exon 2; 1.4% (*n* = 1) for G12S in exon 2; 1.4% (*n* = 1) for G12V in exon 2; and 1.4% (*n* = 1) for G13D in exon 2. The details of *EGFR* and *KRAS* mutations are summarized in Table 3.

**TABLE 3:** Summary of *EGFR* and KRAS mutations.

*EGFR* mutations			
		%	95% CI
Deletion in exon 18	1	1.4	0–7.5
L747P in exon 19	1	1.4	0–7.5
V774M/H773L in exon 20	1	1.4	0–7.5
Insertion in exon 20	1	1.4	0–7.5
L858R in exon 21	1	1.4	0–7.5

KRAS mutations.			
		%	95% CI
G12D in exon 2	3	4.2	0.9–11.7
G12C in exon 2	2	2.8	0.3–9.7
G12S/G12A in exon 2	1	1.4	0–7.5
G12A in exon 2	1	1.4	0–7.5
G12F in exon 2	1	1.4	0–7.5
G12S in exon 2	1	1.4	0–7.5
G12V in exon 2	1	1.4	0–7.5
G13D in exon 2	1	1.4	0–7.5

**FIGURE 3:**
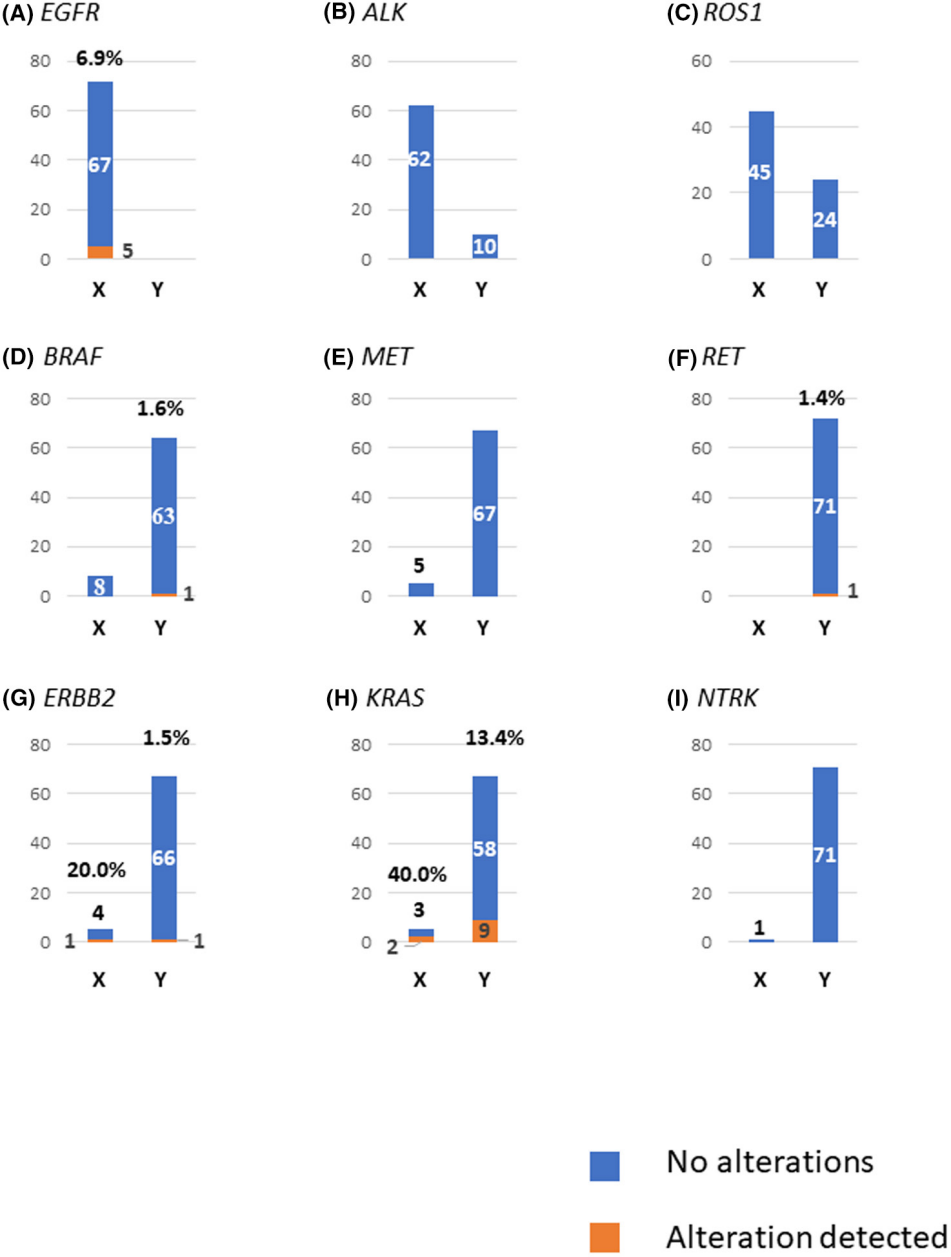
Rate of alterations detected by liquid biopsy after prior negative tissue biopsy (bar X) or no prior tissue test (bar Y). Columns show: (A) *EGFR*; (B) *ALK*; (C) *ROS1*; (D) *BRAF*; (E) *MET*; (F) *RET*; (G) *ERBB2*; (H) *KRAS*; and (I) *NTRK*. Vertical axis represents the number of patients. Blue, no alteration detected; orange, alteration detected.

Among the patients with negative results by single‐plex analysis of each clinically relevant gene, mutation detection rates by comprehensive liquid biopsy were as follows: 40.0% (2/5) for those previously tested for *KRAS*; 20.0% (1/5) for those previously tested for *ERBB2*; 6.9% (5/72) for patients previously tested for *EGFR*; 0% (0/62) for those previously tested for *ALK,* 0% (0/45) for those previously tested for *ROS1*; 0% (0/8) for those previously tested for *BRAF*; 0% (0/5) for those previously tested for *MET*; and 0% (0/1) for those previously tested for *NTRK* (Figure 3, bar X). Mutation detection rates for patients who were not evaluated for specific genes before ctDNA analysis were as follows: 13.4% (9/67) for *KRAS*; 1.6% (1/64) for *BRAF*; 1.5% (1/67) for *ERBB2*; 1.4% (1/72) for *RET*; 0% (0/10) for *ALK*; 0% (0/24) for *ROS1*; 0% (0/67) for *MET*; and 0% (0/71) for *NTRK* (Figure 3, bar Y). No significant differences were identified in the characteristics of patients with gene alterations detected by comprehensive liquid biopsy (*n* = 21) and those without gene alterations (*n* = 51) (Table 4).

**TABLE 4:** Characteristics of patients with (*n* = 21) and without (*n* = 51) clinically informative alterations detected in ctDNA.

	With gene alteration in ctDNA		Without gene alteration in ctDNA		*p*
	*n* = 21	%	*n* = 51	%	
Age (years)					
Mean	71.4		71.4		0.99
Median	72		73		
Range	51–86		47–83		
Sex					
Male	15	71.4	35	68.6	1
Female	6	30.6	16	31.4	
Smoking history					
Never	6	28.5	11	21.6	0.31
Past	11	52.4	26	51	
Current	3	14.3	14	27.4	
Unknown	1	4.8	0	0	
Performance status					
0	6	28.6	18	35.3	0.857
1	13	61.9	29	56.9	
2	2	9.5	4	7.8	
Pathology					
Adenocarcinoma	19	91.7	47	92.2	0.229
Large cell carcinoma	0	0	2	3.9	
Sarcomatoid carcinoma	2	9.5	0	0	
Other	0	0	2	3.9	
Stage					
IIIB	2	9.5	3	5.8	0.797
IIIC	0	0	1	2	
IVA	3	14.3	14	27.5	
IVB	11	52.4	24	47.1	
Postoperative recurrence	4	19	6	11.8	
Post‐chemoradiotherapy recurrence	1	4.8	3	5.8	
Number of genes evaluated by single‐plex test using tissue samples					
1	5	23.8	5	9.8	0.2
2	3	14.3	11	21.6	
3	10	47.6	28	54.9	
4	0	0	4	7.8	
5	0	0	1	2	
6	1	4.8	2	3.9	
7	1	4.8	0	0	
8	1	4.8	0	0	
T stage					
x	1	4.8	1	2	0.901
0	0	0	2	3.9	
1	5	23.8	13	25.5	
2	4	19	10	19.6	
3	7	33.4	12	23.5	
4	4	19	13	23.5	
N stage					
x	0	0	1	2	0.892
0	2	9.5	9	17.6	
1	2	9.5	6	11.8	
2	7	33.4	14	27.5	
3	10	47.6	21	41.2	
M stage					
0	5	23.8	10	19.6	0.981
1a	4	19.1	10	19.6	
1b	2	9.5	7	13.7	
1c	10	47.6	24	47.1	

### 3.4 TAT

**FIGURE 4:**
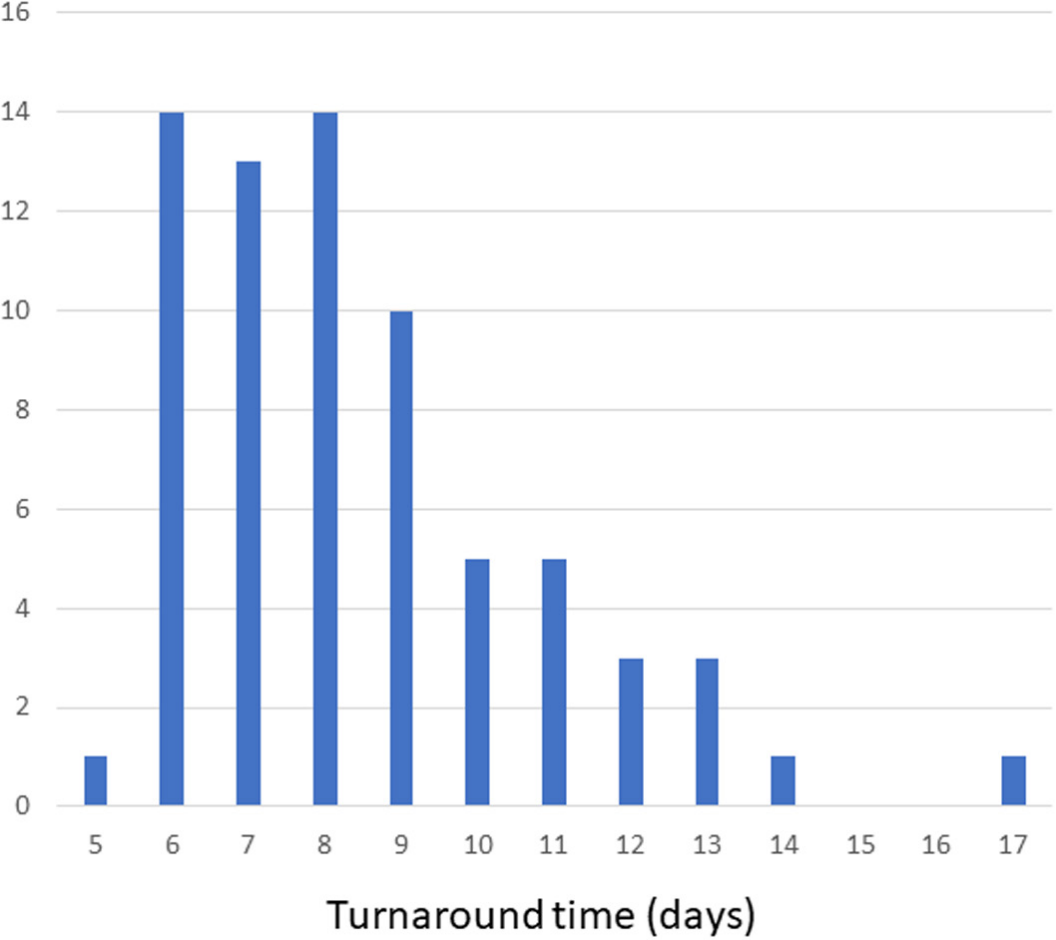
Results for turnaround time. Vertical axis represents the number of patients. One patient required 17 days because improper packaging at the time of shipment required re‐examination.

Median time from the day blood was collected to the day that the result was available was 8 days (range, 5–17 days; Figure 4).

### 3.5 Follow‐up tissue testing

The demographics and test results for the 21 patients for whom driver gene alterations were detected in ctDNA are summarized in Table 5. Among all enrolled patients, four (5.6%; 95% CI, 1.5%–13.6%) underwent re‐analysis of tissue samples. For two patients, this confirmed the presence of driver alterations that were detected in ctDNA (L858R in *EGFR* exon 21 by single‐plex test and V600E in *BRAF* exon 15 by multiplex test). On the other hand, for the two patients in whom *EGFR* exon 18 deletion and exon 20 insertion were detected in ctDNA, the same alteration could not be confirmed by tissue‐based single‐plex test. In another patient without a detectable driver alteration by single‐plex and ctDNA testing, *ALK* fusion was eventually detected by tissue‐based multiplex analysis (Table 5, Patient 22).

**TABLE 5:** Confirmation status of alterations detected in ctDNA analysis.

Patient	Alteration detectedin ctDNA	Mutation type	Singleplex analysisbefore ctDNA analysis	Age (years)	Sex	Smoking status	Pathology	Performance status	T	N	M	Stage	VAF (%)	Companion diagnosis method after ctDNA analysis	Gene detected using companion diagnosis after ctDNA analysis	TKI administered according to companion diagnosis
1	*KRAS*	G12D in exon 2	not investigated	69	female	never	AD	1	T2b	N2	M1c	IVB	0.09			
2		G12D in exon 2	not investigated	72	male	past	AD	1	T4	N2	M1a	postoperative recurrence	0.5			
3		G12D in exon 2	not investigated	67	male	past	AD	0	T3	N0	M1a	IVA	0.2			
4		G12C in exon 2	not investigated	79	male	past	AD	1	T3	N3	M1b	IVB	11.6			
5		G12C in exon 2	not investigated	82	male	unknown	AD	1	T2a	N3	M1b	IVB	18.6			
6		G12A in exon 2	not investigated	72	male	past	SA	0	T1c	N1	M0	postoperative recurrence	3			
7		G13D in exon 2	not investigated	65	female	current	AD	0	T3	N3	M0	IIIB	0.8			
8		G12F in exon 2	not detected	72	male	current	AD	1	T1a	N2	M1c	IVB	1			
9		G12S in exon 2	not detected	60	male	past	AD	1	T3	N3	M1c	IVB	0.07			
10		G12V in exon 2	not investigated	86	male	past	AD	2	T3	N1	M1c	IVB	1.1			
11		G12S/G12A in exon 2	not investigated	77	male	never	AD	0	T1b	N2	M1a	IVA	0.4/0.1			
12	*EGFR*	deletion in exon 18	not detected	62	female	never	AD	1	T1a	N3	M1c	IVB	2.6	multiplex	not detected	afatinib
13		insertion in exon 20	not detected	85	female	past	AD	1	T4	N3	M1c	IVB	0.4	singleplex for *EGFR*, *ALK*, and *ROS1*	not detected	not administered
14		L747P in exon 20	not detected	76	male	past	AD	0	T2a	N3	M1c	IVA	1.3			
15		V774M/H773L in exon 20	not detected	66	male	current	AD	2	T2a	N2	M0	postoperative recurrence	0.5/0.5			
16		L858R in exon 21	not detected	79	male	past	AD	0	Tx	N0	M1a	postoperative recurrence	0.3	singleplex for *EGFR*	L858R in *EGFR* Exon 21	osimertinib
17	*ERBB2*	insertion in exon 20	not investigated	74	male	past	AD	1	T4	N3	M1c	IVB	19.4			
18		insertion in exon 20	not investigated	73	male	past	AD	1	T1	N2	M0	post‐chemoradiotherapy recurrence	47.9			
19		insertion in exon 20	not detected	62	female	never	AD	1	T3	N3	M1c	IVB	1.8			
20	*BRAF*	V600E in exon 15	not investigated	51	male	never	SA	1	T4	N2	M1c	IVB	18.3	multiplex	V600E in *BRAF* Exon 15	not administered
21	*KIF5B‐RET*	fusion	not investigated	71	female	never	AD	1	T3	N3	M0	IIIB	0.7			
22	None	/	not detected	72	female	never	AD	1	Tx	Nx	M1c	postoperative recurrence	/	multiplex	*ALK* fusion	alectinib

Abbreviations: AD, adenocarcinoma; SA, sarcomatoid carcinoma; VAF, variant allele frequency.

### 3.6 Targeted therapy for patients based on ctDNA results

Among all 72 patients, two patients (2.8%; 95% CI, 0.3%–9.7%) were treated by targeted therapies according to ctDNA results. One patient showed L858R in *EGFR* exon 21, which was undetected at first with a tissue‐based single‐plex test using a bone metastasis sample and later confirmed by performing an approved single‐plex test performed on a primary tumor specimen that had been surgically resected 10 years prior. This patient was treated with osimertinib (Table 5, Patient 16). A patient whose tumor showed deletion in *EGFR* exon 18 detected by ctDNA, underwent confirmatory tissue‐based multiplex analysis again, whereas gene alterations were not detected (Table 5, Patient 12). That patient was treated with afatinib and showed partial response with a treatment duration of 6 months. Tyrosine kinase inhibitor (TKI) treatment was not administered in two patients harboring *KRAS* G12C mutations because confirmation by companion diagnosis had not been performed in these patients. Those patients in whom *BRAF* mutation or *RET* fusion was detected were responding to previous treatments and had not been treated with TKIs.

## 4 DISCUSSION

This is the first prospective study to demonstrate the ability of follow‐on ctDNA testing to detect informative genomic alterations in patients with advanced NSCLC for whom initial tumor tissue testing was negative for at least *EGFR* mutations. Using this approach, we identified relevant alterations in 29.2% (95% CI, 19.0%–41.1%) of patients tested. Alterations were identified in some previously tested genes as well as in genes that were not tested, reflecting the challenges of single‐plex biomarker testing in clinical practice. Potentially actionable driver gene alterations, according to standards established in international guidelines, were detected in 16.7% of samples tested (12/72; 12 patients who were excluded had tumors harboring *KRAS* mutations other than G12C). For follow‐on testing, examination of ctDNA was less invasive than histological biopsy. Furthermore, the median time from blood testing to result confirmation tended to be shorter (8 days; range, 5–17 days), against 11 days (range, 8–14 days) associated with one of the tissue‐based multi‐gene assays available in Japan.^14^ Because some previous reports have shown that selecting the appropriate first‐line molecularly targeted therapy leads to benefits in terms of long‐term outcomes,^3^, ^8^ the results of shortening TAT may also be important in clinical settings.

In Japan, the Oncomine Dx Target test Multi CDx System (Thermo Fisher Scientific Inc., Waltham, MA, USA) was approved as a CDx test to identify alterations in five driver genes for first‐line targeted treatments in NSCLC are available: *EGFR*, *ALK*, *ROS1*, *BRAF* (exon 15 V600E), and *RET*. This tissue‐based multiplex assay was studied in the Lung Cancer Genomic Screening Project for Individualized Medicine in Japan (LC‐SCRUM‐Japan). Among 1688 advanced NSCLC patients without *EGFR* gene mutations according to prior testing, the following driver alterations were detected: 17.0% for *KRAS* mutations; 8.4% for *EGFR* mutations; 6.3% for *ERBB2* mutations; 4.2% for *BRAF* mutations; 4% for *ROS1* fusion; 3% for *RET* fusion; 3% for *ALK* fusion; 2% for *MET* exon 14‐skipping mutation; and 1% for the *KRAS* mutations and *BRAF* mutations combined (total rate, 48.9%).^13^ Here, we showed with a comprehensive liquid biopsy and median TAT of 8 days that the total detection rate was 29.2% (*p* = 0.001; 95% CI, 19.0%–41.1%), including some patients who were tested for more than *EGFR* mutations. Although direct comparisons cannot be made because of differences in patient background, study sample sizes and sources of tumor tissue, the detection rates we observed with ctDNA analysis were somewhat lower but confirmed that testing with a platform that did not rely on tissue could identify a clinically meaningful proportion of patients who could be candidates for first‐line targeted therapy.

In our study, *EGFR* mutations were detected in the second highest number of patients (*n* = 5, 6.9%) using ctDNA analysis despite the fact that prior tissue testing did not find *EGFR* mutations. We did not collect information on which assays were used for determining *EGFR*‐negative status. Most single‐plex *EGFR* gene mutation assays focus on common sites of alterations, as so‐called “hot spots,” and do not fully interrogate the gene. Therefore, less common but potentially actionable alterations such as V774M/H773L in exon 20, L747P in exon 19, and exon 18 deletion, as detected in our study, may not be found using currently approved companion diagnostic tests.^15^ Theoretically, the *EGFR* L858R and exon 20 insertion should have been detected by single‐plex testing. The failure of currently available hotspot testing to achieve such detection may be due to tumor sampling and the low proportion of tumor cells in samples. The same reasons were considered for the two patients showing positive results for *KRAS* mutations, and one patient showing a positive result for ERBB2 mutation in ctDNA analysis, with negative results by single‐plex analysis of each clinically relevant gene.

Leighl et al. reported that ctDNA NGS identified eight genomic biomarkers, including *EGFR* mutations, at a rate at least as high as that of tissue genotyping, with high tissue concordance, more rapidly and more completely than tissue‐based genotyping among 282 patients with newly diagnosed metastatic NSCLC.^16^ In addition, Zugazagoitia et al. analyzed the clinical utility of the same assay in patients with inadequate tumor samples for tissue genotyping with 93 advanced‐stage lung adenocarcinoma patients, identifying actionable alterations in 13 patients (14%).^17^Similar to our approach, other researchers have found that plasma ctDNA analysis and tissue genotyping are complementary tools for therapeutic decision‐making for advanced NSCLC.^18^ However, because the ctDNA analysis used in this study has not been approved for first‐line decision making in Japan, the results could not be used for treatment decisions. The approval of ctDNA analysis as a comprehensive genome‐profiling test before first‐line treatment is desired in Japan, but not yet available.

One key limitation in this study was bias in patient selection. When the sample quantity is limited, prioritizing multiplex tissue‐based testing over single‐plex gene analysis is preferred. Since our study excluded patients who had undergone multiplex gene analysis, some patients may have preferentially chosen to undergo single‐plex gene analysis for the purposes of enrolment in the study. The BRAVE study investigated how to select first‐line treatment based on testing of *EGFR*, *ALK*, *ROS1*, and PD‐L1 in nonsquamous NSCLC patients in 11 medical centers in Japan and showed that, while 197 of 202 patients (97.5%) were tested for *EGFR*, only 39.1% were concurrently tested for all three genomic biomarkers.^19^ Another important limitation is the effectiveness of the molecular targeted drug was not confirmed for the patients whose gene alteration was detected. Aggarwal et al. reported the therapeutic effect of molecularly targeted drug in 42 NSCLC patients for whom driver alterations were detected by ctDNA.^20^ According to those results, 36 (85.7%) of the 42 patients with evaluable results achieved either complete response (*n* = 1), partial response (*n* = 19), or stable disease (*n* = 16). Paik et al. also reported similar efficacy in a study using tepotinib for lung cancer patients harboring *MET* exon 14‐skipping mutation between tissue‐positive patients and ctDNA‐positive patients.^21^ These results support the reliable positive percentage agreement of the ctDNA analysis used in our study. Finally, in the developed world, where resources are readily available and reimbursed, the trend is to perform multiplex testing as the initial examination. However, this is not the practice in all situations. In some cases, using a single‐plex examination may be more practical and cost‐effective, such as when gene analysis results need to be obtained quickly or to identify common alterations that account for the majority of actionable biomarkers for which treatments are accessible. For example, in Asia, single‐plex testing for *EGFR* mutation remains an important examination because of the high frequency of *EGFR* mutation. This study was a single‐arm trial with a relatively small sample size and was exploratory in nature. The present results should therefore be confirmed by a large clinical study to evaluate the detection rate for driver gene alterations using cfDNA and a tissue sample.

In conclusion, this multicenter, prospective observational study demonstrated that comprehensive next‐generation sequencing of ctDNA from plasma samples identified driver gene alterations in 29.2% of patients with untreated advanced nonsquamous NSCLC. These results were obtained within a reasonable timeframe. The use of multiplex testing of tissue specimens has become more common for lung cancer, but in situations where the probability of a driver alteration is not rare and the results of initial testing are negative, rapid follow‐on comprehensive liquid biopsy could be useful to ensure that actionable driver alterations missed by initial testing are detected prior to initiation of first‐line treatment for advanced NSCLC.

## AUTHOR CONTRIBUTIONS

**Takehiro Uemura:** Conceptualization (equal); data curation (equal); investigation (equal); visualization (lead); writing – original draft (lead); writing – review and editing (equal). **Hirotsugu Kenmotsu:** Conceptualization (lead); data curation (equal); funding acquisition (lead); investigation (equal); visualization (equal); writing – review and editing (equal). **Daisuke Hazama:** Data curation (equal); investigation (equal); writing – review and editing (supporting). **Shunsuke Teraoka:** Data curation (equal); investigation (equal); writing – review and editing (supporting). **Hiroshi kobe:** Data curation (equal); investigation (equal); writing – review and editing (supporting). **Koichi Azuma:** Data curation (equal); investigation (equal); writing – review and editing (supporting). **Teppei Yamaguchi:** Data curation (equal); investigation (equal); writing – review and editing (supporting). **Takeshi Masuda:** Data curation (equal); investigation (equal); writing – review and editing (supporting). **Toshihide Yokoyama:** Data curation (equal); investigation (equal); writing – review and editing (supporting). **Kohei Otsubo:** Data curation (equal); investigation (equal); writing – review and editing (supporting). **Koji Haratani:** Data curation (equal); investigation (equal); writing – review and editing (supporting). **Daisuke Hayakawa:** Data curation (equal); investigation (equal); writing – review and editing (supporting). **Masahide Oki:** Data curation (equal); investigation (equal); writing – review and editing (supporting). **Shinnosuke Takemoto:** Data curation (equal); investigation (equal); writing – review and editing (supporting). **Tomohiro Ozaki:** Data curation (equal); investigation (equal); writing – review and editing (supporting). **Yusaku Akashi:** Data curation (equal); investigation (equal); writing – review and editing (supporting). **AKITO HATA:** Data curation (equal); investigation (equal); writing – review and editing (supporting). **Hiroya Hashimoto:** Formal analysis (lead); writing – review and editing (supporting). **Nobuyuki Yamamoto:** Supervision (equal); writing – review and editing (supporting). **Kazuhiko Nakagawa:** Supervision (equal); writing – review and editing (supporting).

## CONFLICT OF INTEREST STATEMENT

T. Uemura reports receiving personal fees from Chugai. H. Kenmotsu reports receiving grants from AstraZeneca, Eli Lilly, Novartis Pharma, and Ono Pharmaceutical, and LOXO Oncology. K. Azuma reports receiving personal fees from AstraZeneca, Bristol‐Myers Squibb, Chugai, MSD, and Ono Pharmaceutical, Pfizer, and Takeda. M. Oki reports receiving a grant from AbbVie, Janssen, PAREXEL International Corp., and Sanofi. A. Hata reports receiving grants and personal fees from AstraZeneca, and Eli Lilly; personal fees from Chugai, and Pfizer; and a grant from Boehringer Ingelheim, and MSD. N. Yamamoto reports receiving grants and personal fees from Amgen, AstraZeneca, Boehringer‐Ingelheim, Bristol‐Myers Squibb, Chugai, Daiichi‐Sankyo, Eli Lilly, Janssen, MSD, Nippon Kayaku, Novartis, Sanofi, Taiho, and Tsumura; personal fees from GlaxoSmithKline, Guardant health Japan, Kyorin, Kyowa‐Kirin, Ono Pharmaceutical, Otsuka, Pfizer, Takeda, Merck, and Miyarisan; and grants from AbbVie, Asahi Kasei Pharma, Astellas, Eisai, Shionogi, and Tosoh. K. Nakagawa reports receiving patent royalties from Daiichi‐Sankyo; grants and personal fees from Chugai, and Ono Pharmaceutical; personal fees from Eli Lilly, Merck, and Pfizer; and grants from Bristol Myers Squibb, Covance Japan Inc., Daiichi‐Sankyo, EPS Corporation, Eisai, GlaxoSmithKline, IQVIA Services JAPAN, Janssen, Japan Clinical Research Operations, Medical Research Support, MSD, Novartis, PAREXEL International Corp., PRA Health Sciences Inc., Sanofi, SYNEOS HEALTH CLINICAL, and Sysmex Corporation, Taiho, and Takeda; and scholarship endowments from Chugai, Eisai, Ono Pharmaceutical, and Takeda. The remaining authors declare no conflict of interest.

## FUNDING INFORMATION

This study was funded by Guardant Health AMEA, Inc.

## ETHICS STATEMENT

Approval of the research protocol by an institutional review board: This study was approved by the Research Ethics Committee of each participating institution and was carried out in accordance with the Declaration of Helsinki.

## INFORMED CONSENT

Written informed consent was obtained from all participants.

## REGISTRATION NUMBER OF THE STUDY/TRIAL

UMIN000041583.

## Data Availability

The data that support the findings of this study are available on request from the corresponding author upon reasonable request.
